# Preventing alcohol misuse in young people aged 9-11 years through promoting family communication: an exploratory evaluation of the Kids, Adults Together (KAT) Programme

**DOI:** 10.1186/1471-2458-11-810

**Published:** 2011-10-17

**Authors:** Heather Rothwell, Jeremy Segrott

**Affiliations:** 1 Cardiff Institute of Society and Health, Cardiff School of Social Sciences, Cardiff University, 1-3 Museum Place, Cardiff, UK

## Abstract

**Background:**

Alcohol misuse by young people is an important public health issue, and has led to the development of a range of prevention interventions. Evidence concerning the most effective approaches to intervention design and implementation is limited. Parental involvement in school-based interventions is important, but many programmes fail to recruit large numbers of parents. This paper reports findings from an exploratory evaluation of a new alcohol misuse prevention programme - Kids, Adults Together (KAT), which comprised a classroom component, engagement with parents through a fun evening for families with children aged 9-11 years, and a DVD. The evaluation aimed to establish the programme's theoretical basis, explore implementation processes and acceptability, and identify plausible precursors of the intended long-term outcomes.

**Methods:**

Documentary analysis and interviews with key personnel examined the programme's development. Classroom preparation and KAT family events in two schools were observed. Focus groups with children, and interviews with parents who attended KAT family events were held immediately after programme delivery, and again after three months. Interviews with head teachers and with teachers who delivered the classroom preparation were conducted. Follow-up interviews with programme personnel were undertaken. Questionnaires were sent to parents of all children involved in classroom preparation.

**Results:**

KAT achieved high levels of acceptability and involvement among both children and parents. Main perceived impacts of the programme were increased pro-social communication within families (including discussions about harmful parental alcohol consumption), heightened knowledge and awareness of the effects of alcohol consumption and key legal and health issues, and changes in parental drinking behaviours.

**Conclusions:**

KAT demonstrated promise as a prevention intervention, primarily through its impact on knowledge and communication processes within families, and its ability to engage with large numbers of parents. A key programme mechanism was the classroom preparation's facilitation of parental involvement in the family fun evening. The programme also incorporated features identified in the literature as likely to increase effectiveness, including a focus on harm reduction, interactive delivery, and targeting primary-school-age children. Further research is needed to test and develop programme theory through implementation in different school contexts, and to examine potential longer-term impacts, and the feasibility of large scale delivery.

## Background

This paper reports the findings from an exploratory evaluation of a school-based alcohol misuse prevention programme - Kids, Adults Together (KAT), which engaged with primary school children and their parents/carers. A range of health and social impacts of alcohol misuse by young people has been documented, including disorderly and violent behaviour, risky sexual behaviour [1], accidental injury, poor school attendance and achievement [2,3], and increased risks of alcohol-related problems in later life [4-7]. The global costs of alcohol misuse related to such impacts are high [8], and a World Health Assembly resolution in 1983 called on Member States to prioritise prevention [9]. However, during recent years concern has grown throughout Europe and North America regarding frequent and excessive use of alcohol by young people and early initiation of alcohol consumption [10-15].

In the UK most efforts to prevent alcohol misuse depend on schools as a means of reaching large numbers of young people and, potentially, their families [16,17], and classroom-based education for children is an established part of the curriculum [18,19]. In the UK, external agencies such as DARE (Drug Abuse Resistance Education) also provide school-based substance misuse education, and Life Education Centres (LECs) use mobile units which make brief visits to schools to deliver substance-misuse education. DARE was consistently found to have little or no effect on children's drug use and this was linked to its promotion of abstinence, use of predominantly non-interactive classroom teaching and failure to consider structural influences such as the family on children's reception of programme content [20-24].

The incorporation of activities or materials for parents or the engagement of parents and children in joint activities has been identified as an important aspect of school-based prevention interventions [23,25], driven by the recognition that the family environment plays an important role in shaping young people's attitudes and behaviour towards alcohol, as well as influencing a range of both protective and risk factors [26,27]. The family is an early social influence, particularly on the timing of young people's first alcohol use [28]. Parental norms and examples may encourage children's early alcohol use by providing models of alcohol consumption [29,30], or easy access to alcoholic drinks. While parental rules relating to alcohol are an important factor, broader forms of parental monitoring, and the quality of relationships within families also operate as protective factors [29,31-33]. UK Governments provide strong strategic support for school-based substance misuse education and for prevention initiatives which involve external agencies and children's families, with all governments now expecting schools to engage with the wider community [34-37]. Additionally, most schools in the UK have made a commitment to becoming health promoting schools, which involves linking participation to health [38].

This increased focus on parenting is part of a broader need for more rigorous evaluation of programmes to address young people's alcohol misuse [39-41], including those delivered in schools [42]. However, a number of features have been identified which are likely to increase the effectiveness of interventions. These include a focus on harm reduction rather than abstinence; interactive activities and delivery; targeting children at primary school, when they are less likely to have experimented with alcohol or other substances; and involving parents as well as children [20,22,23,33,43-45]. Whilst the *intention *to involve parents is relatively easy to incorporate into programme designs, the *process *of engaging parents can be complex and challenging [7,16,46,47]. For instance, the Blueprint Drugs Education Programme in England aimed to involve parents [48], but attracted a poor response. It was modified to provide free transport, refreshments, crèche facilities, and gifts, including a DVD, at launch events, but with little success in terms of parental engagement. Lack of parental involvement was sometimes associated with schools with high levels of disadvantage and with secondary schools. Other reasons were lack of appropriate recruitment and publicity, and parents perceiving the programme as irrelevant, not having time to take part, or being unwilling to associate with other parents [47]. An evaluation of 'Unplugged' - a substance abuse prevention intervention delivered in schools across seven European countries - also reported low levels of parental involvement [49,50]. Several other UK prevention projects designed to involve parents have not been well described or evaluated, or have disappeared when short-term funding has ended [51]. In the UK both DARE and LECs have been adapted to include parents [52,53]. Life Education is the only UK programme which appears to have involved significant numbers of parents and has attracted over 10,000 parents to attend parenting programmes [52]. However, there is little analysis of key processes leading to parental participation in Life Education, the precise nature of parents' involvement, levels of participation across schools, and the effect on substance misuse.

The present study aimed to contribute to current research by evaluating the development and early implementation of a new school-based alcohol misuse prevention programme for children aged 9-11 years, comprising a classroom component, engagement with parents through a family fun evening, and a DVD. It aimed to establish the programme's theoretical basis, explore implementation processes and acceptability (particularly in relation to engagement with parents), and identify plausible precursors of the intended long-term outcome which could be used as indicators of likely effectiveness.

## Methods

### Background to the introduction of KAT

KAT was conceived by Substance Misuse prevention workers who perceived that attempts to reduce alcohol misuse by young people had achieved little effect because they did not address the powerful influence of the home environment and parental behaviour. The programme was originally conceptualised as a DVD for parents. However, the organizer, a police officer, subsequently identified the Australian Parents, Adults, Kids Together (PAKT) programme run by Life Education Victoria [54] which had successfully engaged parents, as a model for KAT. PAKT involves primary school children preparing activities to present at a 'family forum' at the school to which they invite their parents, and producing 'take home bags' containing leaflets and other items.

KAT retains the main structure of PAKT, with the addition of a specially made DVD in the 'goody bag' for children to take home, for families to watch together. Both KAT and PAKT programmes are universal and target the whole (school) population, not just those considered to be at increased risk [49,55]. Both address Year 5 and 6 children (aged 9-11 years) and their parents, and take a harm reduction approach. However, the KAT teachers' pack supporting the classroom preparation was developed independently by a multi-agency working group; and KAT deals solely with alcohol, whereas PAKT addresses substance misuse in general.

### Programme development: aims and objectives

The organiser convened a working group to plan the family event and classroom preparation. Aims and learning objectives identified for the classroom component in the teachers' pack are included at Table 1. The organiser identified KAT's long-term aim as reducing the number of young people who drink too much and then become involved in antisocial behaviour and crime; and the short term objective as "for parents and children to openly recognise and discuss the issues." The long-term aim was clear to all the working group members but KAT objectives were not shared among members of the working group and the organiser. Although minutes of a meeting held in June 2007 recorded objectives concerning attitudes, knowledge and skills relating to alcohol use, interviews with five members of the working group suggested that this did not capture how they expected the programme to achieve its aim. Interviewees mentioned a range of short-term objectives and only two mentioned encouraging family communication about alcohol. Other objectives included changes in parents' drinking behaviour and providing help for schools in delivering the PSE curriculum.

**Table 1:** Aims and learning objectives of KAT classroom preparation stated in the Teachers' Pack

**1. Questionnaire**
*Aim: *To establish children's baseline knowledge of alcohol and its misuse.
**2. (Literacy/PSE)**
*Aim: *Alcohol (effects and consequences) theme linked to teaching children useful skills in collecting data
*Learning objective: *To focus on alcohol and its effects and consequences
**3. (Literacy/Art/PSE)**
*Aim: *Alcohol (effects and consequences) theme linked to teaching children skills in art and design
*Learning objectives:*
• To design an alcohol information poster
• To design a poster advertising KAT Family event
• To design an invitation (inviting parents/carers to the KAT Family Event)
• To focus on alcohol and its effects and consequences
**4. (Drama/Role-play)**
*Aim: *To raise awareness of the effects of alcohol within families
*Learning objectives:*
• To raise awareness of the effects of alcohol use in family situations and how this might affect children
• To enhance participants' reflection on these issues and their attitudes and values related to their alcohol use and how this impacts on their families and the wider community.

### Implementation

KAT was piloted in two schools in South East Wales during 2008. Head teachers at the two schools were members of the working group who volunteered to run the programme. The areas served by the schools had substantially more lone-parent households with dependent children than the national average. Percentages of children entitled to free school meals were well above the national average and attendance figures were below the national target. There were marked differences between the schools' ethos, teaching and communication cultures and the head teachers' leadership styles. The socioeconomic and geographical characteristics of the areas served by the schools were also very different. The first (S1) served an area in the South Wales Valleys where mining had been the chief occupation and unemployment had been high since closure of the mine. The other (S2) was in a suburb of a market town in a rural area where farming and tourism were the most important occupations and there were good transport links.

### Data collection

#### Phase 1

Phase 1 investigated how KAT had originated and developed, its relationship to existing evidence and theory, and its aims. Methods used were an analysis of 32 documents meant to provide an 'audit trail' of programme development, and interviews with six members of the working group who had been involved in setting up the programme, the programme organiser and his assistant, the KAT DVD producer and the organiser of the Australian PAKT programme on which KAT was based.

To allow participants to raise topics which had not been anticipated in advance, a semi-structured approach was adopted, with core topics covered in all interviews, and suggested questions and prompts which allowed flexibility in the exact details discussed. Topics covered: development and planning of KAT; its aims, structure and content and how these differed from PAKT; the fit between KAT and other services and policy objectives; future development of the programme; and how the programme aimed to help families. Examples of questions related to this final topic included 'Who is KAT aimed at? (areas/schools/families with particular needs)', 'How do you think KAT will change parents?' (raise awareness/change parenting behaviour/change drinking behaviour)', and 'Do you think that Police involvement in KAT will affect families' willingness to take part?'. Phase 1 also included interviews with the PAKT organiser in Australia and the DVD producer, which covered similar topics but also addressed issues specific to their role, such as the evaluation of PAKT, and the development and intended effects of the DVD.

#### Phase 2

Phase 2 comprised:

• observation of the classroom preparation and KAT family events in two pilot schools (S1 and S2);

• seven focus groups involving 41 children;

• interviews with both head teachers and with teachers who delivered the classroom preparation (n = 6);

• follow-up interviews with the programme organisers (n = 2);

• follow-up interviews with six working group members;

• interviews with twelve parents who attended the KAT family events; and

• a postal questionnaire for parents of all 110 children involved in the classroom preparation.

Observation was conducted by HR, and JS also observed the fun evening at S1. Observation examined adherence to the KAT manual, the use of interactive methods [56], levels of pupil participation, and which concepts, types of knowledge and skills were addressed. Narrative notes were made during each lesson and fun evening.

Interviews with working group members explored whether they felt the KAT approach could be used to involve parents in other topics, and what they thought about further development of KAT. Two of the working group were also asked to comment on the fun evenings, the DVD, and on some of the findings from interviews with parents. The interview with the programme organiser and his assistant covered organisation of the fun evenings, differences and similarities between the two pilot schools, involvement of the working group, and future programme development. Interviews with school staff covered the circumstances leading to their involvement with KAT, classroom preparation, the fun evenings, programme DVD, and how they thought KAT would fit into other schools.

Two rounds of focus groups and parent interviews were conducted: the first interviews and focus groups were conducted between 6 and 23 days after the KAT event at each school (mean interval 14.6 days; median 13 days). The second round took place three months later. The same parents and children took part at both times, apart from four children who were available to attend only one focus group and one parent who was unavailable for a follow-up interview. The purpose of collecting data three months after participation in the programme was to provide a check in case any programme effects were wholly temporary, rather than to make detailed comparisons between the two time points. However, as family communication emerged as a key intermediate outcome, and because participants volunteered unexpected information about alcohol-related behaviour, we discuss differences across time in relation to these issues in more detail.

The first interviews with parents covered the KAT fun evening, goody bag, DVD and class work and how they thought KAT might affect their families in the future. Follow-up interviews explored how much they remembered about KAT, any perceived effects on the family, and their views on schools' role in raising awareness about alcohol. In the first round of focus groups children discussed the KAT class work, fun evenings, goody bags and DVDs and the importance of KAT for themselves and their families. In the follow-up, groups were asked to recollect KAT by writing or drawing something they remembered and were asked whether since the first focus group they had watched the DVD, talked about KAT or perceived any effects on themselves or family members.

A questionnaire was sent to parents of all children in Years 5 and 6 at both schools, asking for their views on the KAT DVD and fun evening and exploring reasons for non-attendance where applicable. Thirty-eight questionnaires were completed and returned from 27 households (24.5% of all households receiving questionnaires).

Tables 2, 3, 4, and 5 give details of methods and participants, and Table 5 summarises numbers of male and female participants in each component of the study. One temporary female teacher taught on one day only and she was not interviewed. Participants' ethnicity was not ascertained but most, if not all, were white British. All pupil participants were aged 9-11 years.

**Table 2:** KAT Working Group, PAKT organiser and KAT DVD producer: Dates of interviews and backgrounds of participants

Date of first interview (Phase 1)	Date of second interview (Phase 2)	Background of interviewee(s) (WG1-WG6)
1/7/08	-	PAKT organiser

2/7/08	12/12/08	Working Group members: Local Authority Substance Misuse Education and NPHS (joint interviews with 2 interviewees)

4/7/08	6/1/09	Working Group member: Community Arts Development

22/7/08	11/12/08	Working Group member: Police

23/7/08	21/1/09	KAT organiser and assistant (joint interviews with 2 interviewees)

24/7/08	12/12/08	Working Group member: Voluntary organisation

3/9/08	16/12/08	Working Group member: National Public Health Service

4/9/08	-	DVD producer

**Table 3:** Phase 2: Data collection at first pilot school (S1)

Method	Dates	Participants	Approximate duration	
Classroom observation	29/9/08 to 21/10/08	Year 5 and 6 classes (54 children)	10-11 hours	

Fun evening observation	22/10/08	Children and families, school staff, KAT organizers	2 hours	

Staff interviews	24/10/08	Head teacher (H1)	20 minutes	
		
		Year 5 and 6 teachers (T1 and T2 - joint interview)	40 minutes	

Focus groups	7/11/08 and 13/2/09		First	Follow-up
			
		FG1 (6 children in first group, 7 children at follow-up)	30 minutes	50 minutes
		
		FG2 (7 children in first group, 5 children at follow-up)	50 minutes	50 minutes
		FG3 (6 children at both times)	40 minutes	40 minutes

Parent interviews	28/10/08 to 4/11/08 (first time); and 28/1/09 to 12/2/09 (follow-up)	6 mothers (M1-M6)	10-35 minutes each	10-20 minutes each

Parent questionnaires	Week beginning 3/11/08	54 households	(17 completed and returned from 12 households)	

**Table 4:** Phase 2: Data collection at second pilot school (S2)

Method	Dates	Participants	Approximate duration	
Classroom observation	12/11/08 to 25/11/08	Year 5 and 6 classes (56 children)	5 hours	

Fun evening observation	26/11/08	Children and families, school staff, KAT organizers	2 hours	

Staff interviews	28/11/08	Head teacher (H2)	40 minutes	
		
		Year 5 and 6 teachers (T4 and T5 - joint interview)	30 minutes	

Focus groups 8 & 9/12/08 and 31/3/09 & 1/4/09			First	Follow-up
		
		FG4 (5 children at both times)	35 minutes	50 minutes
		
		FG5 (5 children at both times)	40 minutes	1 hour
		
		FG6 (5 children in first group, 4 children at follow-up)	1 hour	50 minutes
		
		FG7 (6 children at both times)	1 hour	1 hour

Parent interviews	15/12/08 to 19/12/08 (first time); and 19/3/09 to 6/4/09 (follow-up)	5 mothers and 1 father (first time - M7-M11 and F1); 4 mothers and 1 father (follow-up - M7, M9-M11 and F1))	10-25 minutes each	10-20 minutes each

Parent questionnaire	Week beginning 1/12/08	56 households	(21 completed and returned from 15 households)	

**Table 5:** Numbers of male and female participants involved in each component of the study

Component	Male	Female	Total
Working Group interviews Phase 1 (including programme organiser and assistant, PAKT organiser and DVD producer)	4	6	10

Working Group interviews Phase 2 (including programme organiser and assistant)	3	5	8

Classroom observation - children (approximate numbers of boys and girls)	50	60	110

Classroom observation - teachers	1	4	5

School staff interviews	1	5	6

Parents' interviews Round 1	1	11	12

Parents' interviews Round 2	1	10	11

Focus groups Round 1	12	27	39

Focus groups Round 2	16	22	38

Questionnaire respondents	25	12	37 + 1 unknown

#### Ethical issues

The evaluation was approved by Cardiff School of Social Sciences Research Ethics Committee. Adult participants gave consent for their own and/or their children's participation. Children were provided with age-appropriate information and asked to sign assent forms before each focus group. Teachers were offered the opportunity to refuse consent for classroom observation, with an assurance of confidentiality if they did so.

### Analysis

The study adopted a thematic content analysis approach [57]. Using NVivo 8, a coding framework was devised based on topics and questions in interview schedules and documentary analysis sheets. Coding was intended to identify particular features of the data which could help to meet the aims of the evaluation. To examine acceptability, text was coded relating to each component of KAT (DVD, fun evening, classroom preparation, goody bag). Codes for interviewees' views on substance misuse education in schools and the future of KAT were used to collate text relating to wider acceptability and feasibility. Programme implementation processes were explored by looking for text relating to (1) the background to the introduction of KAT, (2) planning and organisation of KAT, and (3) the working group. To establish the theoretical basis for the programme, some codes identified text relating to the aims and anticipated impacts of KAT as stated by the developers; different participant groups; location; and timing. These groupings facilitated comparisons which helped to discriminate between features of programme and context; to estimate the persistence of any potential short term impacts; and to differentiate acceptability and perceived impacts for different groups of participants.

One interview and one focus group transcript were coded independently by each researcher and then compared, leading to some adjustments to the framework. Important themes relating to KAT's impact on behaviour and family communication were identified early in the analysis and were also assigned codes. SPSS 16 was used to store questionnaire data and produce descriptive statistics.

## Results

### Classroom preparation

Classroom work was guided by a teachers' pack (Table 1). In S1, children in Years 5 and 6 were taught in mixed classes (taught by T1, T2 and T3). A written plan for classroom preparation was shared with the researcher (HR) who was invited to observe any lessons during which KAT preparation took place. At S2 children were taught in two separate year-group classes (by T4 and T5). Teachers worked more independently with each year group and a breakdown in communication meant that T5 had already begun work based on the teachers' pack before T4 was aware that their class would be taking part in the programme and before the researchers found out that the work had started.

Teaching at S1 was carried out over one week, with further time allocated afterwards so that children could finish their work before the KAT event - all together the preparation was spread across just over three weeks. At S2 there was no evidence that classroom work had been jointly planned or written down. Year 5 did the classroom preparation over 3-4 weeks (mainly delivered in an intensive two week period). Year 6 started classroom preparation before Year 5 but no clear picture emerged of how intensively the programme was taught in this class. Every opportunity was taken to observe the classroom preparation - thus observation was carried out at S1 every day during the week the classroom preparation was happening, together with an extra visit to observe the drama rehearsals. At S2, due to communication difficulties or unplanned decisions to deliver KAT preparation, opportunities for observation were fewer. Thus total observation time in S2 was 5 hours 10 minutes - less than the 10 hours 45 minutes' observation in S1.

Teachers at both schools used the pack as a framework but adapted the details. At S1 the drama/role play work was developed into two short plays for presentation at the fun evening and at S2 one of the teachers wrote a song for performance at the fun evening. Interactive methods were used in both schools, including whole-class discussions, group and pair work. Lessons at S2 included more material about the effects of alcohol on society in general than at S1, where activities were more focused on families.

### Fun evening

Fun evenings at both schools included three activities for parents and children, and short performances by the children. Nearly all activities were introduced and led by the programme organiser and for some, prizes were awarded. At both schools the children's class work was on show and a stall providing information about drugs and alcohol was manned by the head of a voluntary organisation supporting families affected by drug misuse.

Forty to fifty adult family members attended at each school - far above the number at most other comparable school events. Most participants were women and included members of the extended family as well as parents. Adults and children worked together in many activities. Most were reported to be parents who usually supported school events. Both head teachers recognised parents with 'drink problems' in the audience. At both fun evenings the presenter talked about the DVD and urged families to go home and watch it together.

### Goody bags

Children were given drawstring 'KAT' bags containing stationery items, the DVD 'Gone', written information including a laminated sheet 'Encouraging Your Children', and a smoothie drink. In general, parents and children were pleased with the bags and for some children they were the best thing about KAT. The DVD 'Gone' was a drama about a family where the parents drank too much at a barbecue in their garden and behaved thoughtlessly. There were scenes showing the father giving a can of beer to his son and treating him roughly. The children ran away during the night to the family's caravan. When the parents woke in the morning they felt increasingly anxious and guilty as they searched for the children. In the meantime, the caravan had been accidentally set alight and the parents arrived on the scene just after the police and fire crews had rescued the children. A voiceover by one child at the end indicated that the parents behaved more responsibly thereafter.

### Acceptability

Overall, KAT achieved high levels of acceptability among children, parents and school staff. Children had enjoyed KAT, particularly the classroom preparation which they described as 'fun' and as different from normal school work. The children also enjoyed learning about issues relating to alcohol. Parent interviewees liked the fun evening, saying it was interesting, non-judgmental and informative. They enjoyed the informality of the evening:

[...] it was a fun event, you know? You can go along to things, can't you, for smoking or whatever and it's going to be really serious and you know, you mustn't do this and you mustn't do that. But it was all fun and everybody was involved and nobody sort of felt the finger pointing at them. (M10 [parent], first interview)

Ten parent interviewees said they would attend another KAT fun evening if it was held again. They thought KAT was a good way of engaging with parents and that it was entirely appropriate that education about alcohol should be delivered at primary school. Responses from 21 questionnaire respondents who had attended the fun evening supported interview data (Table 6). Staff in both schools felt that the fun evening had been delivered in an appealing and non-stigmatising way. T4 and T5 (S2) pointed out that the way in which the evening was promoted as an opportunity for parents to find out what their children had been working on helped avoid a perception that it was designed to lecture parents. Whilst parent interviewees were positive about KAT, they may not have been representative of all the parents who took part. One child in a focus group at S2 said her mother had thought the fun evening was a "waste of time" (FG4) and some questionnaire respondents were not wholly enthusiastic, with five out of twenty-one saying they would not like to go to another KAT event. However, the head at S1 was certain that if parents had not liked the fun evening they would have left. Whilst most school staff and a member of the working group suggested that KAT might not have appealed to all parents who had current alcohol problems, one head teacher felt that such parents were unlikely to have been offended during the fun evening. Most questionnaire respondents (13 out of 17) who had not attended a fun evening indicated that this was due to other commitments, rather than negative perceptions of the event itself (see Table 6).

**Table 6:** Questionnaire responses to: Please read the following sentences about the alcohol awareness evening and tick ONE box on EACH LINE

	Agree		Disagree		Neither agree nor disagree		Not answered		Total
	**S1**	**S2**	**S1**	**S2**	**S1**	**S2**	**S1**	**S2**	

It was a chance to meet up with friends	1	1	4	6	2	6	1	0	21

It was boring	0	1	7	12	0	0	1	0	21

I liked everything about it	5	11	1	1	2	1	0	0	21

It went on for too long	1	1	4	11	2	1	1	0	21

I enjoyed seeing the displays of children's work	6	13	1	0	1	0	0	0	21

The tea and cakes (S1)/refreshments (S2) were good	2	12	0	0	5	1	1	0	21

There was nothing I liked about it	0	1	7	12	0	0	1	0	21

It was fun	6	11	1	2	1	0	0	0	21

It was good to talk openly about alcohol issues	8	13	0	0	0	0	0	0	21

I would like to go to another KAT event	5	9	1	1	2	3	0	0	21

### Potential impacts of KAT

#### Knowledge

Children in all but one (FG6) of the focus groups described having gained new knowledge about alcohol, including the legal framework surrounding alcohol and key government guidelines, such as the rules on and consequences of drink driving. They had also learnt about the physical impacts upon the body of alcohol, how consumption affected individuals' behaviour, and the effects on other people. Much of this knowledge was anchored within descriptions of the particular activities they had undertaken, such as drama performances, or creating posters.

Children in four focus groups (FG1, FG2, FG4 and FG7) believed that their parents had acquired new knowledge as a result of attending the fun evening, and this was mainly conceptualised around their individual drinking practices and awareness of the impacts of alcohol, rather than in terms of broader parental practice or supervision. Children in Focus Groups 1 and 2 thought parents had been surprised by many of the answers to the questions during the fun evening quizzes, and some of the children felt that they had been teaching their parents new information. Knowledge at the fun evening was based largely on what had been learnt in the classroom preparation:

Child 1: I thought it was really good because we found out a lot about alcohol.

Child 2: And it's like teaching parents more about it, and it was really good teaching the parents about it. (Focus group 1, first meeting)

Teachers 1 and 2 felt that the fun evening 'summed up' the work that had been done in class, that the children were keen to show off their knowledge to their parents, and that parents had learnt from their children. They were impressed at how much the children had remembered, and pointed out that much of their knowledge had come from their own research in class, rather than simply being told key facts and figures.

The perceived impact of KAT on parental knowledge was discussed in nine of the twelve interviews conducted with parents (eight mothers and a father). In five of these interviews parents described having acquired new knowledge relating to the effects of alcohol (such as time taken for alcohol to pass through the body), the law on minimum ages of consumption, recommended maximum safe consumption levels, and statistics on the number of young people treated in hospital for alcohol-related injury/illness.

Most of the knowledge acquired by parents appeared to derive from the fun evening, particularly the quizzes and the commentary provided by the compere. One mother neatly captured the way in which her child had shared their learning in class with her:

Parent: I knew a lot of the answers [in the quiz] because my daughter had told me.

HR: What, she'd talked about what she was doing in school had she?

Parent: Yes, and how many units of alcohol you're allowed and um, all the different things, so like I said she has been quite well informed. (M4 [parent], first interview)

#### Attitudes

There was little evidence that involvement in KAT had led to perceived changes in children's attitudes. Participants in the focus groups discussed their attitudes towards alcohol, but these may have pre-dated KAT, or have been created or modified by it. Overall the children held critical attitudes towards alcohol and its effects. Children in four focus groups (FG1, FG4, FG5 and FG7) were generally disapproving of the negative effects of alcohol consumption, and talked about the importance of their not drinking. But at other points the children focused specifically on the idea of limits to safe or acceptable drinking levels/frequency. Children in four focus groups (FG1, FG3, FG4 and FG7) felt there were circumstances in which it would be acceptable for them to drink small amounts of alcohol, such as a sip at Christmas, and/or described enjoying consuming alcohol (or drinks containing alcohol) such as shandy and wine. There was little evidence that KAT had prompted parents to change or adopt new attitudes towards alcohol. Four parents (M2, M4, M5 and M7) who talked of being worried about the dangers of alcohol and the use of alcohol by their children held pre-existing concerns or attitudes. The programme may have reinforced or validated their concerns, but it had not produced a shift in their thinking.

#### Awareness

Some focus group participants described how they had deepened their understanding of alcohol-related issues after taking part in KAT. For instance, they realised that alcohol was not 'just a drink' but could produce certain effects on the human body. They also felt that they had a better understanding of the importance of not drink driving, and the consequences it could have. Some children said KAT had made them think about issues around alcohol they had not considered before (FG7, second meeting).

KAT had impacted on parents' perceived awareness, both in relation to key facts around alcohol (e.g. maximum recommended consumption limits) and in prompting them to think about new issues:

What do I remember? The quiz ... quizzes that asked us questions and they made us very aware of what we didn't know [laughs]. Um, oh, um, the drugs that were on show. Well I've never been involved in drugs so that was quite an eye opener. [...] it made me more aware of what I didn't know, to be honest. (M4 [parent], second interview)

Discussions in four focus groups (FG1, FG2, FG3 and FG7) and interviews with four parents (M1, M6, M8 and M11) suggested that some parents had started to think about their own drinking practices, particularly how drinking alcohol in front of their children could influence them. Six parents (F1, M2, M3, M4, M9 and M10) also felt that the programme had increased their children's awareness of the issues surrounding alcohol. Staff in both schools believed that KAT had increased parents' and children's awareness of some of the main issues relating to alcohol consumption and misuse.

#### Intention

Evidence from participants suggested that KAT had had little perceived influence on intentions regarding future behaviour. Intentions were mentioned by children at four focus groups, by three parent interviewees and by one child during the classroom preparation. When children talked about their own intentions they said they would drink moderately, if at all, when they were older. However, one mother was sceptical about her own children's intentions to abstain from alcohol:

... A couple of times they've come up and said 'We're never having a drink'. I said 'Well no you will... you've got to experiment, all teenagers experiment ...' (M9 [parent], second interview)

A child in one focus group reported her mother's intention to drink less when she went out with friends (FG1, second meeting); one parent interviewee said she would change her own drinking behaviour (M1 [parent], first interview), and another reported her husband's intention to cut down on alcohol (M11 [parent], second interview). Two participants reported intentions unrelated to alcohol - healthy eating inspired by the smoothie recipes (Child in FG 1, first meeting) and not running away from home after seeing what happened to children in the DVD (M1 [parent], first interview).

#### Communication

The KAT programme's most significant and persistent potential impact on communication was its perceived effect on family conversations about parental drinking. Children in four focus groups (1, 3, 6 and 7) who thought their parents drank too much alcohol reported trying to change their parents' behaviour. This was found at both schools, and was reported immediately and three months after the fun evenings:

HR: [...] you said you talked to your parents after the last [focus group]. What sort of things have you talked to them about?

Child: Well, I said that um... well my mother gotta stop keep on drinking .... (Focus Group 3, second meeting)

Children in Focus Group 4 agreed that "If the parents drink too much, it [KAT] will help you". The children's sense of the programme's relevance to their parents also worked the other way: they did not think that they needed to talk to non-drinking parents about KAT. But parents who drank little or no alcohol thought KAT had been useful in bringing forward discussion about a topic which might otherwise not have come up until later on. And in homes where parents and children already had ongoing discussions about alcohol, two parents (M2 and M6) felt that KAT had supported what they were already telling their children.

At both schools the fun evening appeared to have acted as a catalyst for conversations about what children had done in the classroom and activities during the evening. Parents and children helped each other to answer questions and children told their parents about the work on display. For some, conversations about the fun evening activities and topics went on beyond the event, but this seemed to have occurred more often at S1 than at S2. At S1, eight children from all focus groups and three parents reported conversations just after the event, with only one child saying that they had not talked about it afterwards. At S2, four children in two focus groups and two parents said they had talked about the fun evening later on and nine children said that they had not talked about it.

After three months, less difference was found between the schools. Three parents from S2 (F1, M10 and M9) reported that children were remarking on things they might not have been aware of before, such as noticing people who were drunk in the street, and children in two focus groups (1 and 3) at S1 said that they had talked about KAT/alcohol-related topics at home since the first meeting. One mother also noticed a difference in her husband's approach to alcohol issues: "I think he talks about it more openly and it is something that we can sort of chat about now whereas before he might not have done." (M10 [parent], second interview)

The DVD helped to extend the influence of the programme beyond the school-based components. Children in two focus groups (1 and 2) at S1 said they had been keen to watch it and for friends and family members to join them; and two parents (M5 and M6) reported that their children had watched the DVD more than once. One child had left the DVD ready for her parents' friends to watch when they visited her home (M1). The children had talked at home about what happened in the DVD, how alcohol could affect people, or just whether they had enjoyed it or not. During all the focus groups children discussed the DVD story at length, giving opinions on the characters and their behaviour, and it was clear that those who had not watched it had had the story explained to them and were able to join in conversations about it. All questionnaire respondents who had watched the DVD (n = 14), from both schools, said they had talked about it afterwards.

The classroom preparation appeared to be effective in promoting communication about alcohol issues amongst members of the class, and five parents (M1, M6, M7, M8, and M9) said their children had talked about it at home. However, two parents (F1 and M10) reported that children had 'mentioned' the class work but no more, and five parents (M2, M3, M5, M7 and M11) said their children had said nothing to them. A child in one focus group (FG7) said they had deliberately 'kept it quiet' so that it would be a surprise at the fun evening. Most children were very keen to go to the fun evening, to show off their work, to see what it was like and to enjoy the refreshments and entertainment. Four parents (M1, M4, M7 and M10) said their children had put pressure on them to attend:

I went along because [child] was saying 'We're having this evening, you've got to come, Mam'. Otherwise I might not have gone because personally I wouldn't have felt I needed to be aware of alcohol because I'm very aware of it. (M4 [parent], second interview)

Children in four focus groups talked about having 'made' or 'forced' their parents to go, and two parents (M8 and M9) who did not seem to have been pushed into it said they went simply because their children were keen to go.

#### Behaviour

Whilst KAT originated in concern about the number of young people misusing alcohol, many focus group discussions revealed children's concern about adult drinking behaviour and that they thought one of the good things about KAT was its potential to reduce the number of adults misusing alcohol. Straight after the fun evening changes in parental behaviour were discussed by children in all focus groups at S1. Six children talked about favourable changes which they perceived to have resulted from KAT. One said that "My dad used to drink a bit, he used to have a couple of cans a night, but he only has like two now, or something like that." (Focus Group 2, first meeting) Another child said her grandfather had started to drink less since she had talked to him about the bad effects of alcohol.

The laminated sheet 'Encouraging your children' had affected some parents' behaviour and it was interesting that this child interpreted 'listening' to mean listening to his concerns about the amount they were drinking:

HR: And (4), what do you think, have your parents been listening to you more since they read it?

Child: Yeah, they, they drank less at night now. (FG1, first meeting)

Three months later, parents' drinking behaviour was discussed again at all the focus groups in S1, and also by one group (Focus Group 7) at S2. Four children reported favourable changes in parental drinking behaviour. However not all parents had responded. Two children reported no change, one commenting "They still don't listen to me."

Children in one focus group also talked about their own experience of different types of alcoholic drinks and one pupil said she had changed her behaviour by diluting the alcohol strength of the shandy she drank (Focus Group 3, second meeting). However, other children in the same group did not seem to have reflected on their own alcohol consumption or experienced any increased parental limitations.

Evidence of perceived behaviour change also came from parents. Two mothers talked about this during the first interviews, and four during the second. One felt that KAT had had a lasting effect on her and the DVD had played a part in this: "Because it does frighten me, especially with the DVD when you think you know that you have gone to bed and the kids are, you know... I have cut down with my drinking." (M11 [parent], Second interview) However, others said there had been no impact on their own (M3 [parent], second interview), their partner's (M5 [parent], second interview) and their teenage son's (M7 [parent], first interview) immoderate drinking.

## Discussion

The findings from this study suggest that KAT has significant potential as an alcohol misuse prevention intervention, through impacting on knowledge and communication processes within the family. Specifically the programme demonstrated strengths in relation to four important features identified in the literature as likely to increase its effectiveness [23,33,43-45].

Firstly KAT engaged **large numbers of parents **- something which many comparable interventions have found challenging [7,23,46,47,58]. The strong connection between the classroom preparation and the fun evening appeared to be a key mechanism. There was a forward movement throughout the class work powered by the children's desire to present their work to their families and for some, the hope that parents and other family members would drink less alcohol if the children could pass their knowledge on to them. Classroom work operated as a component of KAT and as part of the process of engaging parents in subsequent programme activities. The effectiveness of 'pupil pester power' has been noted as an example of good practice in encouraging parents' attendance at school social events [59], and KAT, while not directly advocating 'pestering', taps directly into children's need for their efforts to be recognised by their parents.

Secondly, KAT adopts a **harm reduction approach**, a key feature of which involves mitigating the negative consequences of alcohol misuse in ways which are compatible with individual needs [44]. Acquiring knowledge and awareness through participation in KAT seems to have enabled parents and children to make a range of decisions about alcohol which fitted their own circumstances. Parents may be less likely to teach children about the health implications of alcohol misuse because the latter are less likely than social consequences to be part of their own experience [60]. The finding that many parents recalled information about the effects of alcohol on internal organs and the negative consequences on health suggests that KAT may have increased parents' capacity to guide and inform their children. Whilst KAT was not designed to address alcohol-misuse problems, there was evidence that it could reach families with such problems and raise awareness of the need for change in ways which participants found helpful. As a universal prevention intervention KAT should operate as part of an integrated package of services offered in schools, and by more specialist agencies.

KAT facilitated both the acquisition of knowledge and awareness of how knowledge could be applied to deal with issues which were important to individual participants, partly because its aims and target audience were open to multiple interpretations by different groups. Some participants saw the fun evening primarily as an education event for children, for instance, whilst others viewed it as mainly providing information for parents. KAT was able to communicate messages to parents in an indirect way, either by presenting the information as something that the children were learning about, or constructing the fun evening as an event where parents came to find out what their children had been doing.

Thirdly, KAT used **interactive activities and delivery techniques**. Children enjoyed the classroom preparation and said it was fun, even though the content appeared to have raised concerns for some about the welfare of family members who were perceived to drink excessively. They learned quickly through drama, discussion, practical activities and use of computers because these activities were enjoyably different from their normal lessons. Children worked with the idea that the knowledge was intended for them to pass on to others through the posters, plays and other work prepared for the fun evening. Thus they urged their parents to attend the fun evening, and at the event they were keen to talk to them about what they had done and to show off their knowledge by supplying answers to the quiz questions and "teaching their parents". So what they learned through interactive methods was itself the foundation of further interaction.

Although parents felt they had learned things through attending the fun evening, this had been achieved as part of their entertainment, by taking part in activities which did not expose or assume ignorance about alcohol issues. At the same time, attendance at the event demonstrated that they were responsible parents who supported their children's education. Some parents favourably contrasted the style of presentation with other 'lecturing' approaches they had experienced and activities at the fun evening overcame the awkwardness some parents may feel when talking to their children about alcohol consumption [61].

Fourthly the programme **targeted primary school aged children**, and thus initiated family communication about alcohol at a stage when parents are still a primary point of attachment [62]. Previous research suggests that parents who postpone discussion of alcohol-related issues may find their timing unpropitious. Young people typically begin to experiment with alcohol or to consume significant quantities of alcohol in early adolescence [28,63,64] and prevention measures are more effective when targeted at pre-adolescent children.

KAT should be seen as a 'complex intervention', with outcomes derived from the interaction of components rather than each component fulfilling a separate function [65]. Much of the learning in KAT is constructed as the sharing of knowledge between parents and their children. The classroom preparation building towards the fun evening appears to be a crucial mechanism in securing parental participation. The fun evening, DVD and classroom work could be discussed at home, thus extending the influence of the programme beyond the school setting. We also found that KAT had the potential to reach beyond parent-child relationships to larger networks of families and friends who were invited to the fun evening or to watch the DVD in family homes. An established programme run every year in the same school could achieve a cumulative effect, particularly for families with more than one child, with annual events acting as 'booster sessions'.

There were many differences between the pilot schools, including general ethos, class composition, organisation of classroom preparation and content of the fun evening. However, no evidence was found of important differences between participants from each school in terms of KAT's acceptability or its overall potential impact on family communication. The difference in reported communication immediately following the fun evening at each school is difficult to explain but this did not appear to lead on to differences in communication patterns three months later.

Our findings suggest that family communication should be reaffirmed as the main intermediate outcome of KAT, and this is consistent with the Social Development Model [62] which links family communication with children's alcohol-related behaviour later in life. The model hypothesises that the family environment for children's social development incorporates both risk and protective factors which explain children's later pro-social or antisocial behaviour. Patterns of alcohol use may be learned through interaction with parents, and this interaction develops a parent-child bond which facilitates reinforcement of young people's behaviour patterns by parental sanctions or encouragement [62]. In terms of the model the pro-social activities offered to KAT participants open up opportunities for parents and young people to talk about issues relating to alcohol and create an expectation that they will participate jointly. Through such joint interactions parents can reinforce and reward pro-social behaviour in relation to alcohol consumption, potentially leading to parent-child bonding which encourages the children to adopt the beliefs and norms of their parents [62]. However, the beliefs and norms of parents may not always be pro-social. The SDM describes a similar pathway leading to antisocial behaviour by which parents may 'train' children to behave in antisocial ways [66]. According to the SDM, children are more likely to use alcohol in antisocial ways if they become attached to parents who are committed to harmful alcohol use. KAT's effectiveness in the long term therefore may depend upon parents having pro-social values which can be communicated to their children. Reports that parents changed their alcohol-related behaviour in pro-social ways after participating in KAT are thus very interesting. However further research would be needed to explore the psychological and theoretical processes which might be involved.

The above findings should be interpreted in the context of the study limitations. KAT was run as a pilot to establish its feasibility and acceptability and the evaluation was designed to identify potential outcomes and causal mechanisms, rather than measure changes in pre-specified outcomes. Interview and focus-group data are derived from a small sample which may not be representative of the general population. The response rate to the questionnaire was low and there may have been overlap between questionnaire respondents and interviewees. Data on persistence of programme effects between the first and second rounds of interviews and focus groups should be treated with caution because the research process itself may have reinforced and reminded participants of KAT.

The research was not able to explore the in-depth experiences of those parents who did not take part in KAT. Seventeen questionnaire respondents had not attended the fun evening and responses from 13 suggested this was because they had other commitments, not because of objections to an alcohol-related event (Table 6). However, there may have been others who did not take part because they felt uncomfortable about their own drinking behaviour. No systematic data were collected on parents' current or past alcohol-related problems (although some participants volunteered information). Therefore we are unable to relate findings to parents' experience of use/misuse of alcohol.

The way in which KAT acted to engage parents through the children's enthusiasm to go to the fun evening may also have missed those who were least likely to interact positively with their children and were unresponsive to any pressure to attend. Besides parents, other relatives and friends attended the fun evenings and it was not possible to estimate the exact proportion of families represented. It is also important to note that participation (both in relation to the programme and the research) was higher among mothers than fathers.

## Conclusions

The findings from this study suggest that KAT merits further research and development because it incorporates significant features already known to increase the effectiveness of prevention interventions, it has the potential to create a surprisingly big impact through the synergy of its components, and it fits well within behavioural development theory. In terms of the MRC framework for trials of complex interventions [65], the present study represents the modelling stage. Progressing to an exploratory trial would provide opportunities to test and develop programme theory through implementation in a range of school contexts, and to examine potential longer-term impacts, the feasibility of large scale delivery, and resources and structures needed. Findings support retention of the overall programme design, especially the connection between classroom preparation and fun evening which was the key to the high level of parental participation.

Future programme evaluation should be designed to identify, verify and explore evidence of parental behaviour change following participation in the programme. This has implications for programme theory, which may require revision to incorporate explanations of parental, as well as children's behaviour. Given the finding that some children became concerned about family members' alcohol consumption, future programmes should ensure appropriate support is available for children taking part and children's views on the effectiveness and acceptability of support services should be explored. Finally, further research should assess programme reach, especially in relation to fathers, and families where alcohol problems exist, and the extent to which KAT can influence parents with anti-social norms. These questions highlight the complexity of engaging parents in school-based prevention interventions and the value of shaping family communication processes in such interventions. The findings from this study suggest that KAT may have the potential to address both parental engagement and family communication by attending to the connections between them.

## Competing interests

The authors declare that they have no competing interests.

## Authors' contributions

JS was the principal investigator, and primarily responsible for the research design.

HR was primarily responsible for data collection. Both authors contributed to data analysis. HR drafted the article, and both authors contributed to critically revising the manuscript for important intellectual content. Both authors read and approved the revised manuscript.

## Pre-publication history

The pre-publication history for this paper can be accessed here:

http://www.biomedcentral.com/1471-2458/11/810/prepub
